# Year-round dengue fever in Pakistan, highlighting the surge amidst ongoing flood havoc and the COVID-19 pandemic: a comprehensive review

**DOI:** 10.1097/MS9.0000000000000418

**Published:** 2023-04-10

**Authors:** Shehroze Tabassum, Aroma Naeem, Abubakar Nazir, Farhan Naeem, Saima Gill, Shehram Tabassum

**Affiliations:** a King Edward Medical University, Lahore; b Faisalabad Medical University, Faisalabad, Pakistan

**Keywords:** dengue fever, infectious disease, Pakistan, public health

## Abstract

Dengue fever (DF) is an arthropod-borne viral infection caused by four serotypes of dengue virus (DENV 1–4) transmitted to the host by the vector mosquito *Aedes*, which causes fever, vomiting, headache, joint pain, muscle pain, and a distinctive itching and skin rash, ultimately leading to dengue hemorrhagic fever and dengue shock syndrome. The first case of DF in Pakistan was documented in 1994, but outbreak patterns began in 2005. As of 20 August 2022, Pakistan has 875 confirmed cases, raising alarming concerns. Misdiagnosis due to mutual symptoms, lack of an effective vaccine, the weakened and overburdened health system of Pakistan, irrational urbanization, climate change in Pakistan, insufficient waste management system, and a lack of awareness are the significant challenges Pakistan faces and result in recurrent dengue outbreaks every year. The recent flood in Pakistan has caused massive destruction, and stagnant dirty water has facilitated mosquito breeding. Sanitization and spraying, proper waste management, an adequate and advanced diagnostic system, control of population size, public awareness, and promotion of medical research and global collaboration, especially amidst flood devastation, are recommended to combat this deadly infection in Pakistan. This article aims to comprehensively review the year-round DF in Pakistan, highlighting the surge amidst ongoing flood havoc and the coronavirus disease 2019 pandemic.

## Introduction

Highlights

- Dengue fever is an arthropod-borne viral infection transmitted to the host by the vector mosquito *Aedes*, which can lead to death when complicated by dengue hemorrhagic fever and dengue shock syndrome.
- The Pattern outburst began in 2005, and since then it has become endemic to Pakistan. This year’s massive floods in Pakistan are aggravating the already worsening dengue situation in Pakistan
- Our article provides a comprehensive review of the past and present status of dengue, the past and present hurdles faced by Pakistan, the efforts made by the government to mitigate the situation in the past, and the counteractive measures taken to control this deadly infection.

Dengue fever (DF) is an arthropod-borne viral disease caused by four serotypes of dengue virus (DENV 1–4), transmitted to the host by the vector mosquito *Aedes* 1. Symptoms include fever, vomiting, headache, joint pain, muscle pain, and a distinctive itching and skin rash that usually begin 3–4 days after infection. In rare cases, the disease develops into a severe dengue hemorrhagic fever, resulting in bleeding, a low platelet count, and blood plasma leakage, or a dengue shock syndrome in which blood pressure becomes critically low^2^,^3^. The bite of a mosquito vector bearing dengue virus facilitates dengue virus entry white blood cells by binding to them, causing the production of interferons and cytokines, which are responsible for many of the disease symptoms. Due to increased capillary permeability, blood leaks from the blood vessels into the body cavities, causing a decrease in blood flow and ultimately decreasing blood pressure, due to which vital organs suffer. The platelet count also decreases, increasing the bleeding risk^4^.

Direct and indirect methods are used for diagnostic purposes. Direct methods include virus isolation, RT-PCR, and dengue nonstructural protein one antigen detection. Indirect methods include serological tests, that is, immunoglobulin (Ig)G and IgM detection. Direct methods are preferred during the first 7 days after the onset of fever. Virus isolation and RT-PCR have largely been replaced by the rapid diagnostic detection of nonstructural protein one antigen^2^. If more than 7 days have passed, serologic tests are used to detect IgM. The presence of IgM confirms a recent infection. The detection of IgG can be due to a recent or past infection. Therefore, serial titers are required to confirm the diagnosis^5^.

## Comparing the past and current status of dengue in Pakistan

**Figure 1:**
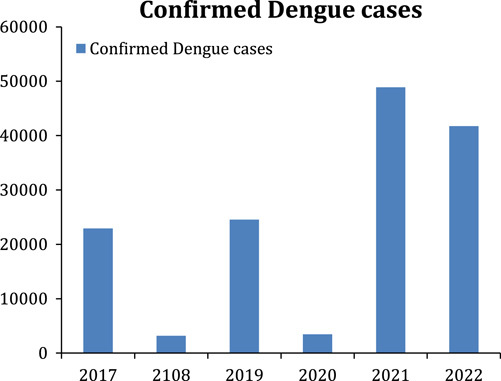
Illustrates the number of confirmed dengue cases in Pakistan.

**Figure 2:**
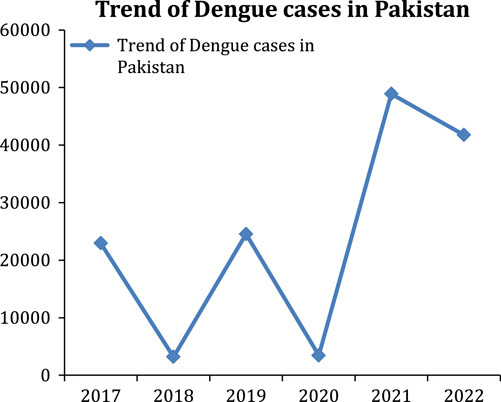
Illustrates the trend of dengue cases in Pakistan.

The earliest outbreak of dengue dates from 1779, but the details about its viral cause, spread, and complications were comprehended in the early 20th century. DF has become an international issue since the Second World War. It is prevalent in 120 countries, especially in Southeast Asia and South America^6^. Approximately 390 million cases of DF are reported annually, but only 96 million are clinically evident. A population of more than 3 billion is at risk of getting this viral infection^7^. Every year, Pakistan reports hundreds of cases of DF. Most of the cases are reported from the southeast of the country. Lahore and the twin cities (Rawalpindi and Islamabad) are significantly affected by it. As of 20 August 2022, Pakistan has reported 1807 suspected cases with 875 confirmed cases. There had been an increase of 932 suspected cases during the past 1.5 months^8^, raising alarming concerns. A DF outbreak occurs every year in Pakistan. It has become a national risk amidst the coronavirus disease 2019 (COVID-19) and monkeypox pandemics. According to the WHO, the first case of DF in Pakistan was registered in 1994. However, the yearly outburst pattern started in Karachi in November 2005. Before 2006, dengue was limited to certain areas of Pakistan. Since 2010, Pakistan has been affected by annual dengue outbreaks, which peak in the postmonsoon season^9^. According to the National Institute of Health, about 22 938 DF cases were reported in 2017. More than 3200 in 2018, 24 547 cases in 2019, and 3442 cases were reported in 2020. In 2021, a significant rise in cases was seen in Lahore and the twin cities (Rawalpindi and Islamabad), where the confirmed cases were reported to be 48 906. The outbreak came to its seasonal end in the winter, though some cases were reported until December^10^. This year, Pakistan is again facing a dengue outbreak, and cases are increasing daily, with a total of 41 746 confirmed cases reported as of 11 October 2022^8^. Figures 1 and 2 show the number of confirmed dengue cases and the trend of dengue cases in Pakistan, respectively, during recent years in Pakistan^8^,^10^.

## Past and present obstacles in battling dengue in Pakistan

### Misdiagnosis due to mutual symptoms

**Figure 3:**
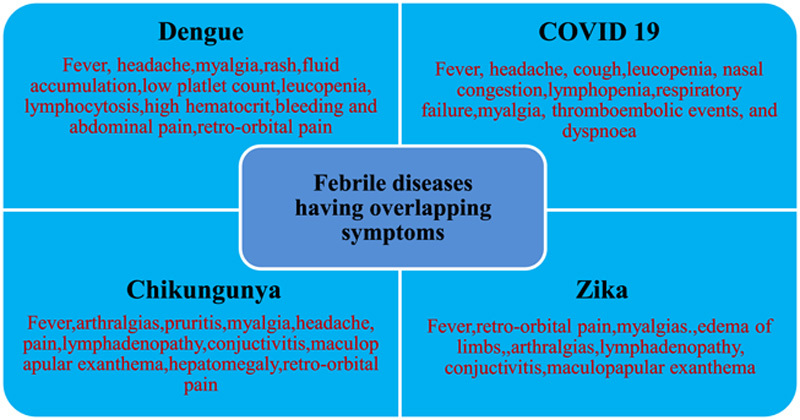
Illustrates overlapping symptoms of dengue with other febrile diseases. COVID-19, coronavirus disease 2019.

Since the symptoms of DF are similar to those of other febrile viral diseases, it becomes difficult to accurately diagnose dengue clinically. Patients presenting with complaints of fatigue, fever, skin rashes, muscle aches, and petechiae with unremarkable chest radiographs, suggesting severe viral diseases, make it quite difficult to distinguish between dengue and COVID-19^11^. A comprehensive comparison between dengue and COVID-19 is written in Table 1 12. Similarly, Zika and Chikungunya also have similar signs and symptoms and are strong differentials of dengue. Overlapping symptoms of Dengue, COVID-19, Chikungunya, and Zika are illustrated in Figure 3.

**Table 1:** Comparison between dengue and COVID-19.

Features	Dengue	COVID-19
Causative agent	Dengue virus (4 serotypes)	SARS-CoV-2
Transmission	Vector: infected *Aedes* species mosquito	Through respiratory droplets
Incubation period	3–10 days	4–14 days
Clinical course	Mild to critical	Mild to critical
Signs and symptoms	Mild disease: fever severe headache with pain behind eyes Nausea and vomiting Muscle aches and body pain Leukopenia Rash Severe disease: hepatomegaly, abdominal pain and tenderness, ascites, mucosal bleeding	Mild to moderate disease Fever Cough Lethargy Chills Shortness of breath Body aches Headache Loss of taste and smell Runny nose Diarrhea, nausea, vomiting, sore throat
Complications	Dengue shock syndrome, dengue hemorrhagic fever, fluid accumulation causing respiratory distress, liver disease, heart complications	Respiratory failure, shock, multiorgan dysfunction

COVID-19, coronavirus disease 2019; SARS-CoV-2, severe acute respiratory syndrome coronavirus-2.

### Lack of effective vaccine

There has yet to be an effective vaccine developed for dengue, one of the significant challenges developing countries like Pakistan face, resulting in thousands of dengue cases each year. Sanof-Pasteur developed a tetravalent dengue vaccine that has been allowed for patient usage, but its efficacy has put a question mark on it^13^. The disease is treated mainly by managing the symptoms that occur during its course. Moreover, the health system of Pakistan lacks adequate funds for research to develop a vaccine for such diseases.

### Susceptibility to all serotypes and secondary infections

There are four different serotypes of dengue virus (DENV 1–4). An infection with one serotype provides long-lasting immunity to the homologous serotype but not to other serotypes. DF is endemic in Pakistan, with widespread distribution of all serotypes, and the population is at risk of re-infection. Due to secondary infections, people are more susceptible to developing severe dengue that requires hospital admissions. Consequently, catastrophic sequelae can occur if such cases are not promptly and effectively treated^14^.

### The overburdened health system of Pakistan amidst the COVID-19

The health care system is overburdened and facing a financial crisis as well. The health system is not strong enough to deal with outbreaks like dengue amidst COVID-19. Most cases in remote areas of Pakistan remain unreported due to a lack of testing facilities. Moreover, health care facilities are more oriented toward fighting COVID-19, which may cause many dengue cases to go unreported^9^.

### Irrational urbanization

Unplanned urbanization is associated with dengue spread through different socioeconomic factors, including population growth, sewage issues, and access to reliable water sources^2^.

### Climate changes in Pakistan

The spread of the dengue virus is inversely proportional to the rise in temperature. The majority of cases in Pakistan are reported from July to September. Heavy rainfall, ideal temperature, and humidity during this period provide convenient circumstances for the survival, growth, and breeding of *Aedes* mosquitoes. The climate of Pakistan is a major factor contributing to the recurrent outbreaks of dengue in Pakistan^15^,^16^.

### Insufficient waste management system

Waste management authorities fail to fulfill their responsibilities by adequately managing the waste, thus polluting the environment. Sewage waste and open drainage systems can act as a habitat for the dengue virus, thus catalyzing the spread of the virus^17^.

### Lack of awareness

There is a lack of understanding of the causes and complications of DF in the general population of Pakistan. Children are not appropriately educated in schools regarding its symptoms and preventive measures. Moreover, most people in endemic areas do not use mosquito nets or mosquito repellants for protection.

## Massive floods aggravating the dengue situation in Pakistan

As of June 2022, rainfall in Pakistan was 2.9 times higher than the previous 30-year average. Since 1961, Pakistan saw its wettest August in August 2022. The most severely affected province was Sindh. With 33 million people affected, the ensuing floods have injured 13 000 people and claimed about 2000 lives^18^. Thirteen million homes were damaged, and 800 000 houses were completely destroyed. More than 2 million individuals were left without a place to live^19^. Tentative analyses show that the current level of destruction is significantly worse than that brought on by previous floods, including the devastating floods of 2010^20^.

Before the heavy rain and ensuing floods, Pakistan had already documented a considerable number of dengue cases. The condition is worsened by the flood, especially in camps and places where water and sanitation infrastructure has been devastated. Standing water serves as an ideal breeding place for mosquitoes that spread dengue^20^. The correlation between record-breaking rainfall in August 2022, followed by the highest number of dengue cases in October 2022 nationwide, seems obvious. The incidence of dengue infection in Pakistan appears to be directly correlated with the amount of precipitation, and dengue infections were most prevalent in the areas that experienced flooding^21^. Pakistan is already afflicted with waterborne disease outbreaks recently^22^,^23^; the present dengue situation, exacerbated by floods, can prove a threat to Pakistan’s public health and safety.

## Efforts done by the Government of Pakistan for dengue control in past years

### Punjab province

In Punjab province, *Aedes* larvae surveillance and space spraying operations are being carried out as part of integrated vector management (IVM). Rawalpindi and Islamabad city have undergone fumigation and spot checks since a field hospital in Lahore opened. To contain the dengue outbreak in Islamabad, multisectoral coordinated activities have been started, including vector surveillance, breeding site destruction, the active finding of cases and contacts, patient management, waste management, and space and indoor residual spraying^14^.

### Sindh and KPK province

In Sindh province and 10 Union Councils of the Peshawar district in the Khyber Pakhtunkhwa (KPK) province, control activities and vector surveillance are now being conducted as part of a special campaign^14^.

### Balochistan province

In Balochistan province, some districts underwent selective indoor residual spraying operations. At specific hotspots in the Kech District, space spraying operations and breeding site management are conducted ad hoc^14^.

WHO and other international organizations are helping Pakistan by strengthening surveillance and reporting systems, ensuring the availability of logistics required for diagnosis, treatment, and control (pesticides, nets, etc.) of dengue, and training health care providers and surveillance teams, including entomologists^14^.

## Counteracting measures needed to eradicate dengue in Pakistan

Although it is commendable that the Government of Pakistan has taken practical steps to prevent and control dengue. But there is still a dire need for specific measures to be taken to alleviate the burden of DF in Pakistan.

### Vector control measures

WHO encourages IVM, which is a strategic method to manage mosquito vectors. To eliminate potential breeding places, lower vector populations, and reduce personal exposure, IVM actions should be effectively implemented. This should include methods for protecting individuals and homes and tactics for controlling vector larvae and adults (such as source reduction, chemical control measures, and environmental management). All sites where there is human-vector contact (places of residence, workplaces, schools, and hospitals) should be the focus of vector control initiatives. Weekly draining, cleaning, and covering of household water storage containers must be done in this regard. If mosquito bites occur indoors, skin creams or mosquito repellant sprays should be, and household insecticide aerosol products should be encouraged^14^. The municipal committee must be vigilant in the rainy seasons to avoid stagnant water, which can provide a medium for vector growth. District management must prioritize filling stagnant water reservoirs and demoralize the concept of open water containers. The government must dispose of the garbage and wastes properly to maintain a healthy environment and control the spread of dengue in the community.

### An adequate and advanced diagnostic system

Although diagnostic facilities are available in the country’s main areas, the facilities are not uniformly distributed and are lacking in various underprivileged areas. The government must organize a proper diagnostic system for testing dengue in remote areas to keep the viral load in check before it gets too late to handle the case with limited resources. Moreover, the laboratory testing facilities should be equipped with advanced technologies like PCR and antibody testing to distinguish between dengue and related diseases. Similar to other infectious diseases, there must be early case detection, prompt case isolation, quick traceback, close monitoring of those at risk, appropriate personal protection, and safe burial, especially in underprivileged areas.

### Control in population size

The population size must be controlled as it will lower the burden on Pakistan’s already weakened health care system, enabling it to provide better and extraordinary services to the people affected by DF. This is crucial for a developing country like Pakistan where massive population size and irrational urbanization have already crippled the country.

### Public awareness

Health education is a fundamental part of any infectious disease control program. People can better comprehend the dengue virus by being aware of the vector’s life cycle, ecology, and biology, which encourages healthy behaviors^10^. Awareness sessions must be conducted throughout the country to make people aware of their responsibilities as citizens to combat recurrent dengue outbreaks. The teachers, elders, and influential people should be sensitized about the severity of the virus^24^. Both electronic and print media should be engaged to play their part.

### Promotion of medical research and global collaboration

Extensive research should be carried out in the area of vaccine and antiviral development for dengue. The development of an effective vaccine can work wonders in eradicating dengue not only from Pakistan but from the whole world. Therefore, international collaboration is also essential in this cause.

## Emergency preventive measures in recent flood-affected areas

The current floods in Pakistan have posed severe health risks for the affected population. The current situation provides favorable conditions for dengue to spread. The situation should be controlled by an appropriate and immediate action plan. Prevention at this stage should be taken before things get out of hand. Restoration of the drainage system would help decrease mosquitoes’ breeding places. People should have access to safe drinking water and health care facilities. Other priorities include strengthening and expanding disease surveillance, preventing and controlling outbreaks, and ensuring a well-coordinated response at the national and subnational levels, with the coordination of all relevant partners. The government should allocate an appropriate budget for this cause. National and international nongovernment organizations can also contribute to the motivation to make it more effective.

## Conclusion

Dengue is a significant challenge for the health care system of many under-developed countries, especially Pakistan, affecting many people each year. Pakistan is experiencing major public health threats in the face of the current massive flood that facilitates the transmission of this deadly disease. Almost one-third of Pakistan’s population is suffering from life-threatening infections, including cholera, malaria, typhoid, etc., due to flood consequences. Although the government of Pakistan has initiated plans to tackle the problem, there is still a need for improvement. Diagnostic and treatment facilities should be extended in far-flung rural areas, and measures should be taken to destroy breeding places for mosquitoes that transmit the disease. Strict standard operating procedures must be formed and implemented effectively to prevent this year-round fatal disease in Pakistan.

## Ethical approval

Not required.

## Consent

Not applicable.

## Source of funding

None.

## Author contribution

S.T.: conceptualization. S.T., A.N., A.N., F.N., S.G., and S.T.: writing. S.T. and S.G.: review with critical comments. S.T. and S.G.: editing.

## Conflicts of interest disclosure

None declared by any authors involved in the manuscript.

## Research registration unique identifying number (UIN)

None.

## Guarantor

Shehroze Tabassum.

## Provenance and peer review

Not commissioned, externally peer reviewed.

## References

[1] Guzman MG Harris E . Dengue. Lancet 2015;385:453–465. 25230594 10.1016/S0140-6736(14)60572-9

[2] Dengue and severe dengue. Accessed 6 January 2023. https://www.who.int/news-room/fact-sheets/detail/dengue-and-severe-dengue

[3] Dengue fever | The BMJ. Accessed 6 January 2023. https://www.bmj.com/content/351/bmj.h4661.long

[4] Martina BEE Koraka P Osterhaus ADME . Dengue virus pathogenesis: an integrated view. Clin Microbiol Rev 2009;22:564–581. 19822889 10.1128/CMR.00035-09 PMC2772360

[5] Muller DA Depelsenaire ACI Young PR . Clinical and laboratory diagnosis of dengue virus infection. J Infect Dis 2017;215(suppl 2):S89–S95. 28403441 10.1093/infdis/jiw649

[6] Gubler DJ . Dengue and dengue hemorrhagic fever. Clin Microbiol Rev 1998;11:480–496. 9665979 10.1128/cmr.11.3.480 PMC88892

[7] Bhatt S Gething PW Brady OJ . The global distribution and burden of dengue. Nature 2013;496:504–507. 23563266 10.1038/nature12060 PMC3651993

[8] European Centre for Disease Prevention and Control. Dengue worldwide overview. Accessed 6 January 2023. https://www.ecdc.europa.eu/en/dengue-monthly

[9] Yousaf A Khan FMA Hasan MM . Dengue, measles, and COVID-19: a threefold challenge to public health security in Pakistan. Ethics Med Public Health 2021;19:100704. 34230890 10.1016/j.jemep.2021.100704 PMC8249682

[10] ReliefWeb. Pakistan: Dengue Response – Final Report, DREF Operation No. MDRPK022 – Pakistan. Accessed 7 January 2023. https://reliefweb.int/report/pakistan/pakistan-dengue-response-final-report-dref-operation-ndeg-mdrpk022

[11] Joob B Wiwanitkit V . COVID-19 can present with a rash and be mistaken for dengue. J Am Acad Dermatol 2020;82:e177. 32213305 10.1016/j.jaad.2020.03.036 PMC7156802

[12] CDC. Dengue or COVID-19 | CDC. Centers for Disease Control and Prevention. 2020. Accessed 7 January 2023. https://www.cdc.gov/dengue/healthcare-providers/dengue-or-covid.html

[13] Guy B Briand O Lang J . Development of the Sanofi Pasteur tetravalent dengue vaccine: one more step forward. Vaccine 2015;33:7100–7111. 26475445 10.1016/j.vaccine.2015.09.108

[14] Dengue fever – Pakistan. Accessed 7 January 2023. https://www.who.int/emergencies/disease-outbreak-news/item/dengue-fever-pakistan

[15] Khalid B Ghaffar A . Dengue transmission based on urban environmental gradients in different cities of Pakistan. Int J Biometeorol 2015;59:267–283. 24817491 10.1007/s00484-014-0840-6

[16] Environmental risk factors and hotspot analysis of dengue distribution in Pakistan – Climate Change and Human Health Literature Portal. Accessed 7 January 2023 https://tools.niehs.nih.gov/cchhl/index.cfm/detail/10994

[17] Khan J Khan I Amin I . A comprehensive entomological, serological and molecular study of 2013 Dengue Outbreak of Swat, Khyber Pakhtunkhwa, Pakistan. PLoS One 2016;11:e0147416. 26848847 10.1371/journal.pone.0147416 PMC4746065

[18] NIH. Anemia – hemolytic anemia | NHLBI. Accessed 7 January 2023 https://www.nhlbi.nih.gov/health/anemia/hemolytic-anemia

[19] HI. Updates: Pakistan flood response. Accessed 7 January 2023 https://www.hi-us.org/pakistan-updates

[20] Administrator. Major health risks unfolding amid floods in Pakistan. World Health Organization – Regional Office for the Eastern Mediterranean. Accessed 7 January 2023. http://www.emro.who.int/pak/pakistan-news/major-health-risks-unfolding-amid-floods-in-pakistan.html

[21] Abid MA Abid MB . Climate change and the increased burden of dengue fever in Pakistan. Lancet Infect Dis 2023;23:17–18. 36480945 10.1016/S1473-3099(22)00808-8

[22] Tabassum S Naeem A Iqbal H . Cholera outbreak amidst an economic crisis and Covid-19 pandemic in Pakistan. Ann Med Surg 2012;81:104374. 10.1016/j.amsu.2022.104374 PMC9428832 36060438

[23] Tabassum S Naeem A Gill S . Increasing cases of Naegleria fowleri during the time of COVID 19; an emerging concern of Pakistan. Int J Surg Lond Engl 2022;105:106881. 10.1016/j.ijsu.2022.106881 PMC9444334 36075555

[24] PRMP. Dengue Epidemic Prevention, Control & Management Program (DEPCAM). Accessed 7 January 2023. https://prmp.punjab.gov.pk/depcam

